# Pramipexole prevents ischemic cell death via mitochondrial pathways in ischemic stroke

**DOI:** 10.1242/dmm.033860

**Published:** 2019-08-29

**Authors:** Syed Suhail Andrabi, Mubashshir Ali, Heena Tabassum, Sabiha Parveen, Suhel Parvez

**Affiliations:** 1 Department of Medical Elementology and Toxicology, School of Chemical and Life Sciences, Jamia Hamdard, New Delhi 110062, India; 2 Division of Basic Medical Sciences, Indian Council of Medical Research, Ministry of Health and Family Welfare, Government of India, V. Ramalingaswamy Bhawan, New Delhi 110 029, India; 3 Department of Communication Sciences and Disorders, Oklahoma State University, Stillwater, OK 74078, USA

**Keywords:** Dopamine receptor, tMCAO, Mitochondria, Neuroprotection, Neurological recovery

## Abstract

A dopamine D2 receptor agonist, pramipexole, has been found to elicit neuroprotection in patients with Parkinson’s disease and restless leg syndrome. Recent evidence has shown that pramipexole mediates its neuroprotection through mitochondria. Considering this, we examined the possible mitochondrial role of pramipexole in promoting neuroprotection following an ischemic stroke of rat. Male Wistar rats underwent transient middle cerebral artery occlusion (tMCAO) and then received pramipexole (0.25 mg and 1 mg/kg body weight) at 1, 6, 12 and 18 h post-occlusion. A panel of neurological tests and 2,3,5-triphenyl tetrazolium chloride (TTC) staining were performed at 24 h after the surgery. Flow cytometry was used to detect the mitochondrial membrane potential, and mitochondrial levels of reactive oxygen species (ROS) and Ca^2+^, respectively. Mitochondrial oxidative phosphorylation was analyzed by oxygraph (oxygen electrode). Western blotting was used to analyze the expression of various proteins such as Bax, Bcl-2 and cytochrome *c*. Pramipexole promoted the neurological recovery as shown by the panel of neurobehavioral tests and TTC staining. Post-stroke treatment with pramipexole reduced levels of mitochondrial ROS and Ca^2+^ after ischemia. Pramipexole elevated the mitochondrial membrane potential and mitochondrial oxidative phosphorylation. Western blotting showed that pramipexole inhibited the transfer of cytochrome *c* from mitochondria to cytosol, and hence inhibited the mitochondrial permeability transition pore. Thus, our results have demonstrated that post-stroke administration of pramipexole induces the neurological recovery through mitochondrial pathways in ischemia/reperfusion injury.

## Abstract (precis)

**Summary:** Pramipexole produced neurological recovery through mitochondrial-mediated survival pathways in ischemic stroke.

## INTRODUCTION

Stroke is one of the leading causes of death and disability worldwide. Ischemic stroke is characterized by a sudden disruption of blood flow to the brain, causing cell death and damage, which leads to neurological impairments (Patel and McMullen, 2017). In the current scenario, only one drug, tissue plasminogen activator (tPA), is approved that is used in a clinical setting, and new therapies that confer ischemic neuroprotection are desperately needed (Nagy and Nardai, 2017). A paucity of the available treatment options makes the efficacy of this drug applicable only to less than 10% of stroke patients (Alderazi et al., 2012). Stroke causes death of brain cells leading to loss of neurological abilities, including speech, movement and memory, controlled by the affected brain areas (Jokinen et al., 2015). The main goal of the treatment after a stroke should be aimed at neurological recovery and possible prevention from future strokes. The complicated interrelated events triggered by the energy depletion have a specific spatial and temporal pattern arching from the initial damage to the final events of brain repair (Nagy and Nardai, 2017). Incentive methods aimed at anti-apoptotic mechanisms and the augmentation of post-ischemic brain repair could be beneficial. As a result, these processes can be targeted much longer after cell necrosis in the acute and post-acute phases (Christophe et al., 2017). The early loss of neurons results from a primary ischemic injury that triggers a wave of calcium (Ca^2+^) signaling, activating redox mechanisms and downstream signaling cascades. A second progressive phase of ischemic injury occurs during the subacute period. Damaged brain cells that survive the initial ischemic insult go on to experience a delayed neuronal damage that could be salvaged therapeutically (Kalogeris et al., 2016). Mitochondrial dysfunction aggravates ischemic neuronal injury through activation of various molecular mechanisms that is particularly intensified after reperfusion. A normal calcium level is vital for physiological processes; mitochondrial dysfunctions occur through calcium overload in neurons under pathological conditions. An increase in intracellular calcium level in mitochondria triggers the opening of the mitochondrial permeability transition pore (mtPTP) and overproduction of reactive oxygen species (ROS) (Bhatti et al., 2017). Targeting mitochondria could therefore be the promising therapeutic alternative for salvaging progressive ischemic injury.

Over the last decade, there has been stagnation in the number of multinational drug trials for stroke patients due to failure of translational efficacy of the preclinically tested drugs (Minnerup et al., 2012). Repurposing of safe older drugs provides a lower-risk alternative and is thus a novel strategy for improving stroke therapy (Gumbinger et al., 2014). During the past several years, a rising pressure on the pharmacological industry due to the ‘innovation gap’ of rising development costs and stagnant product output have become major reasons for the growing interest in drug repurposing. Among the existing drugs, FDA-approved drug pramipexole (PPX) has been found to be safe and well tolerated by clinical subjects (Hall et al., 1996).

Dopamine agonists have been extensively used as monotherapy in early PD and as adjunctive therapy in advanced stages of PD to improve motor symptoms. Non-ergot dopamine agonists (NEDAs) are now most widely used as they have least adverse effects, similar to the derivatives of ergot (derivatives of ergotamine dopamine agonist). PPX, one of the NEDAs with a high affinity for the D2 subfamily of dopamine receptors, is now available as an extended-release formulation and provides a more continuous therapeutic effect over 24 h (Lauterbach, 2016). PPX has been used to produce neurological recovery in restless leg syndrome (RLS) patients as well (Winkelman et al., 2016). Currently, attention has been drawn towards possibly attaining neuroprotection by inhibiting cell death of the apoptotic brain because of its mitochondria-mediated actions. Since the brain is the organ system that is most reliant on mitochondrial energy supply, it is particularly susceptible to mitochondrial dysfunction (Wallace, 2005). The release of apoptotic factors is related to the mtPTP (Carraro and Bernardi, 2016). One of the early, potentially pathological, mitochondrial responses to Ca^2+^ overload that occurs in cerebral ischemia is the release of cytochrome *c* (cyt c) (Jordan et al., 2011). The released cyt c, along with apoptosis activating factor-1 (Apaf-1) and ATP, activates caspases proteases via apoptosome formation, thereby triggering cellular self-destruction (Shakeri et al., 2017). A few reports in the past have shown that PPX has the potential to inhibit the mtPTP in isolated mitochondria (Cassarino et al., 2000). PPX has been found to be neuroprotective by inactivating the Ca^2+^-triggered mtPTP (Sayeed et al., 2006). Therefore, we examined the ability of PPX to promote recovery in ischemic stroke through the mitochondrial pathway.

To investigate the possible mitochondrial effect of PPX in stroke, we used a transient middle cerebral artery occlusion (tMCAO) model of stroke for this study. We presumed that PPX, having potent anti-apoptotic and anti-oxidative properties, could be a promising drug-repurposing candidate. The neuroprotective effects elicited by PPX have directly been associated with antioxidant effects, mitochondrial stabilization or induction of the anti-apoptotic Bcl-2 family proteins. To examine this, we performed a wide array of experiments to study the efficacy of PPX in a stroke model. We determined the efficacy of neuroprotection by studying motor function restoration and biochemically assessing modulation of mitochondrial dysfunction, including inhibition of mtPTP opening, reduction of oxidative stress and decreased apoptotic mechanism. To the best of our knowledge, this is the first report that discusses treatment with PPX to promote neurological recovery through mitochondria in ischemic stroke of rat.

## RESULTS

### Determination of effective dose and reduction of infarct size

**Fig. 1.**
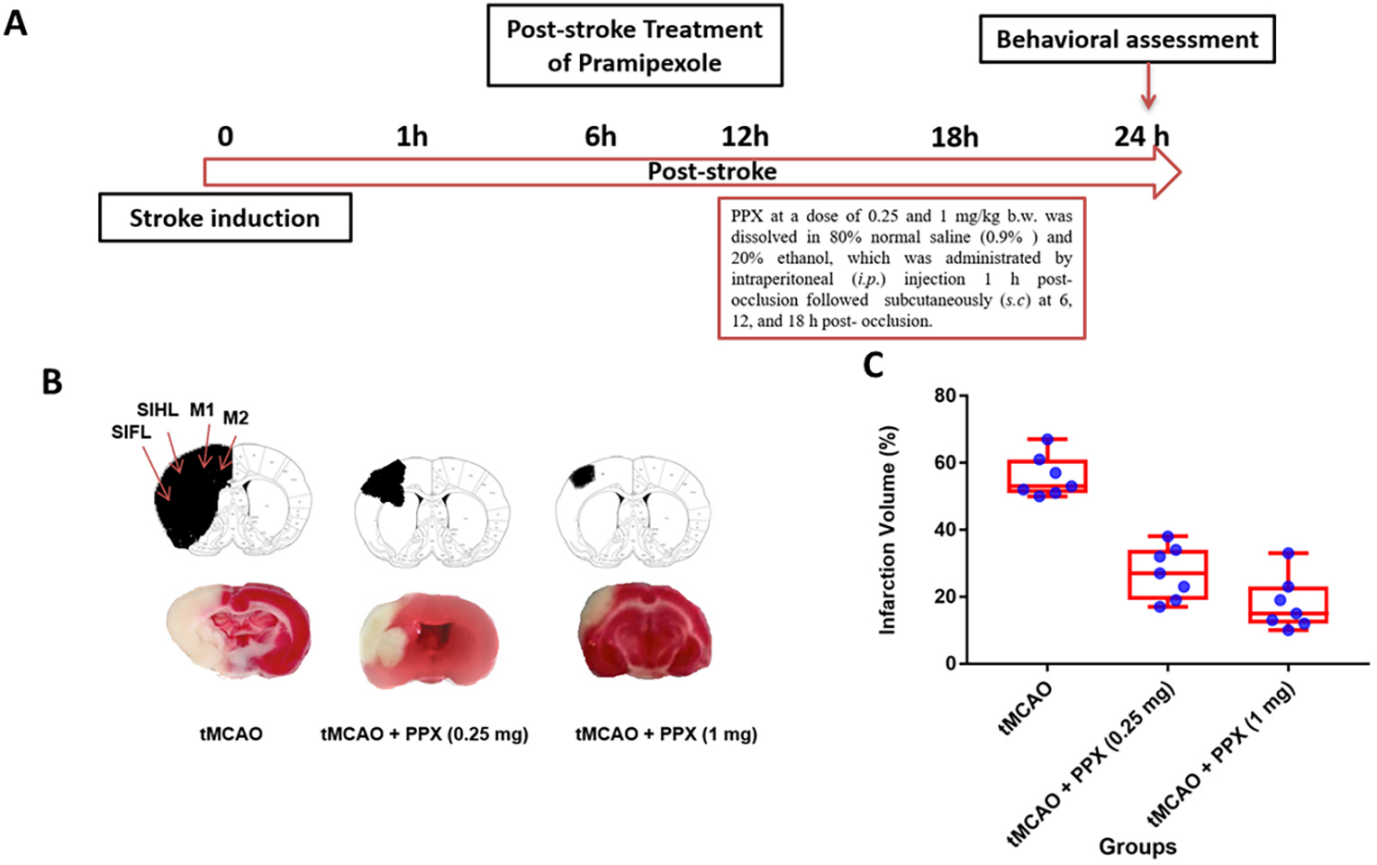
**PPX reduces the infarction volume.** (A) Experimental design. (B) The comparative TTC-stained brain coronal sections of tMCAO- and PPX-treated rats. This shows the lesioned areas in the primary (M1) and secondary (M2) motor cortices of the rat brain. SIFL, primary somatosensory cortex, forelimb region; SIHL, primary somatosensory cortex, hindlimb region. (C) Infarct volume at 24 h post-occlusion. PPX 0.25 and 1 mg/kg b.w. decreased the infarct size (*P*<0.05 and *P*<0.001, respectively).

For all experiments, we followed the methodological standards for performing our stroke experiments (Dirnagl, 2006). Figure 1A shows the treatment schedule. Figure 1B shows the representative infarct size in brain sections stained with TTC in sham, tMCAO-operated and tMCAO plus PPX-treated groups. We tested clinically relevant doses for PPX [0.25 and 1 mg/kg body weight (b.w.)] (Luo et al., 2016). PPX-treated animals show decreased infarction volume as compared to tMCAO-only animals (Fig. 1C). There was a significant difference (*F*_3,18_=49.82, *P*=0.001) between groups. PPX significantly reduced the infarction volume in animals treated with 0.25 mg/kg b.w. (*P*<0.05) and 1 mg/kg b.w. (*P*<0.001), as compared to tMCAO-only animals.

### Effect of PPX on neurological deficits after ischemic stroke

**Fig. 2.**
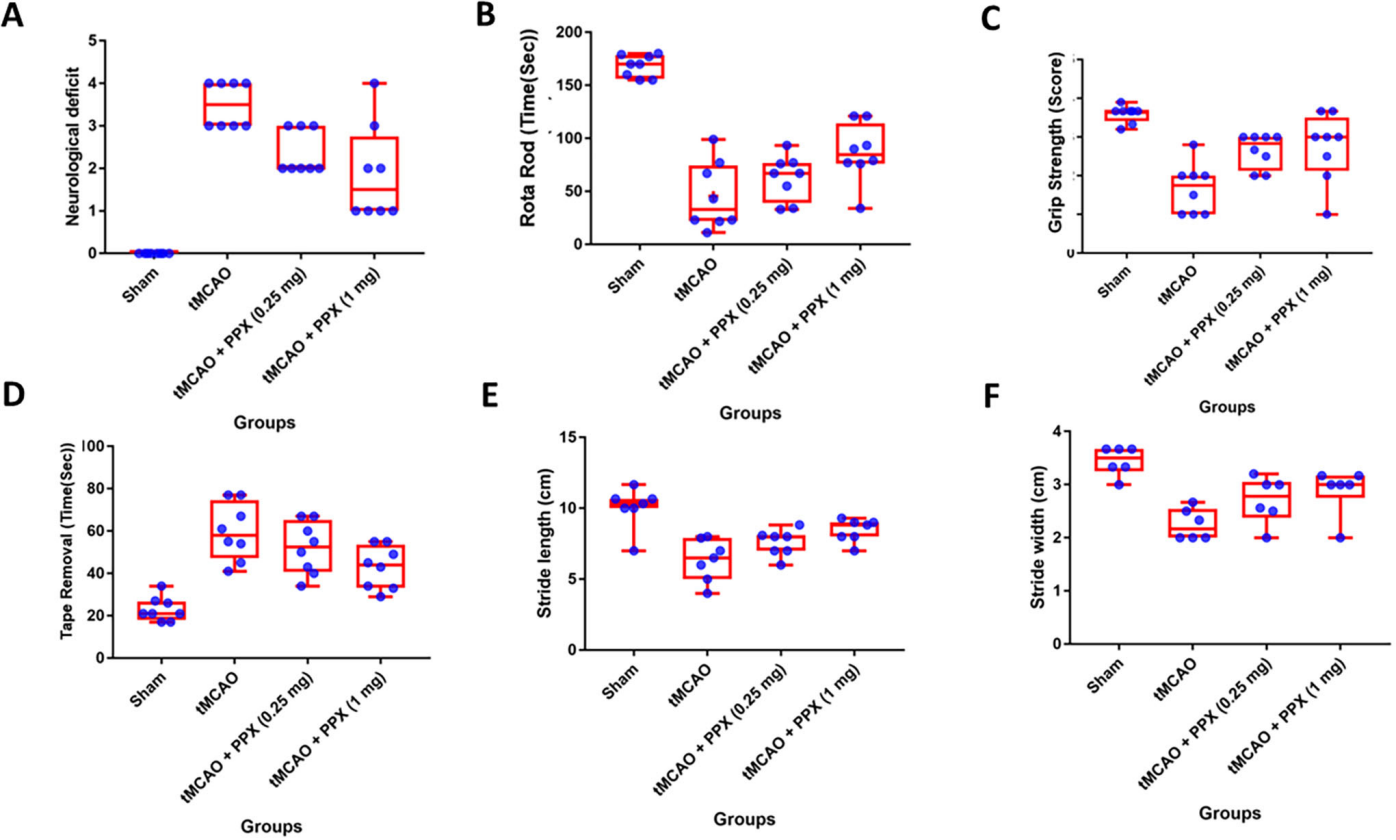
**PPX improves neurological recovery.** (A) Neurological deficit: the rats subjected to tMCAO showed significant (*P*<0.001) neurological deficits as compared to sham animals. PPX-treated rats showed a significant decrease in neurological deficit with both dose regimens (0.25 mg/kg b.w. *P*<0.01, 1 mg/kg b.w. *P*<0.001) as compared to tMCAO-only rats. (B) Rotarod: the rats subjected to tMCAO showed significantly (*P*<0.001) less rotometric performance as compared to sham rats. PPX-treated rats showed a significant increase in latency to fall off the rotarod in a dose-dependent manner (0.25 mg/kg b.w. *P*<0.05, 1 mg/kg b.w *P*<0.01). (C) Grip strength: the rats subjected to tMCAO showed significantly (*P*<0.001) lowered grip strength scores as compared to the sham group. PPX-treated rats showed a significant increase in grip strength score with both doses (0.25 mg/kg b.w. *P*<0.05, 1 mg/kg b.w. *P*<0.01). (D) Tape removal: the rats subjected to tMCAO showed a significant (*P*<0.001) decrease in somatosensory function as compared to sham animals. A higher dose of PPX (1 mg/kg b.w.) decreased the time taken to remove the sticky tape (*P*<0.05) as compared to tMCAO-only rats, but there was no improvement in the group treated with 0.25 mg/kg b.w. (E) Stride length (SL): the rats subjected to tMCAO showed a significant (*P*<0.001) decrease in SL as compared to the sham group. PPX administration caused a significant increase in SL with 1 mg/kg b.w. (*P*<0.001) as compared to tMCAO-only rats. No difference was found between the rats treated with 0.25 mg/kg b.w. as compared to tMCAO-only rats. (F) Stride width (SW): tMCAO led to a significant (*P*<0.001) decrease in SW as compared to sham-treated rats. PPX-treated rats showed a significant increase in SW with only the higher dose (1 mg/kg b.w. *P*<0.05) as compared to tMCAO-only rats.

The neurological impairment was apparent 24 h after tMCAO as shown by various functional outcomes (Fig. 2). PPX treatment produced neurological recovery that is depicted in these behavioral tests (Fig. 2). The neurological deficit showed a significant difference (*F*_3,28_=24.59, *P*=0.001) between groups (Fig. 2A). The PPX-treated rats showed less neurological deficit as compared to tMCAO-only rats at both dose regimens [0.25 and 1 mg/kg b.w. (*P*<0.05 and *P*<0.01); see Materials and Methods].

### Effect of PPX on motor impairment

In the rotating-beam behavior test, there was a significant difference (*F*_3,28_=40.22, *P*=0.001) between groups (Fig. 2B). PPX treatment increased the time on the rotarod with both doses [0.25 mg/kg b.w. (*P*<0.05) and 1 mg/kg b.w. (*P*<0.01)].

### Effect of PPX on grip strength

Specific to grip strength, there was a significant difference (*F*_3,28_=13.69, *P*=0.001) between groups (Fig. 2C). PPX improved the grip strength in treated animals [0.25 mg/kg b.w. (*P*<0.05) and 1 mg/kg b.w. (*P*<0.01)] (Fig. 2C).

### Effect of PPX on adhesive tape removal

Because stroke affects the somatosensory cortex, the adhesive tape removal test was used to monitor functional recovery as it is used as an indicator for forelimb sensorimotor function. The time taken for the animals to remove the adhesive tape on the affected forelimb (contralateral) increased and it was observed to be maximum at 24 h post-stroke (Fig. 2D). There was a significant difference (*F*_3,28_=17.04, *P*=0.001) between groups (Fig. 2D). Post-stroke treatment with PPX (1 mg/kg b.w.) meant that rats took less time to remove the tape (*P*<0.05). However, there was no significant effect with the lower dose of 0.25 mg/kg b.w. (Fig. 2D).

### Gait analysis

The measures of stride length (SL) showed a significant difference (*F*_3,28_=11.62, *P*=0.01) between groups (Fig. 2E). In addition, the measure of stride width (SW) showed a significant difference (*F*_3,28_=10.60, *P*=0.001) between the groups (Fig. 2E). PPX treatment improved the SL and SW as compared to the tMCAO-operated group only with the 1 mg/kg b.w. dose regimen (SL, *P*<0.05; SW, *P*<0.05; Fig. 2E,F).

### Effect of PPX on mitochondrial health

#### Mitochondrial ROS

**Fig. 3.**
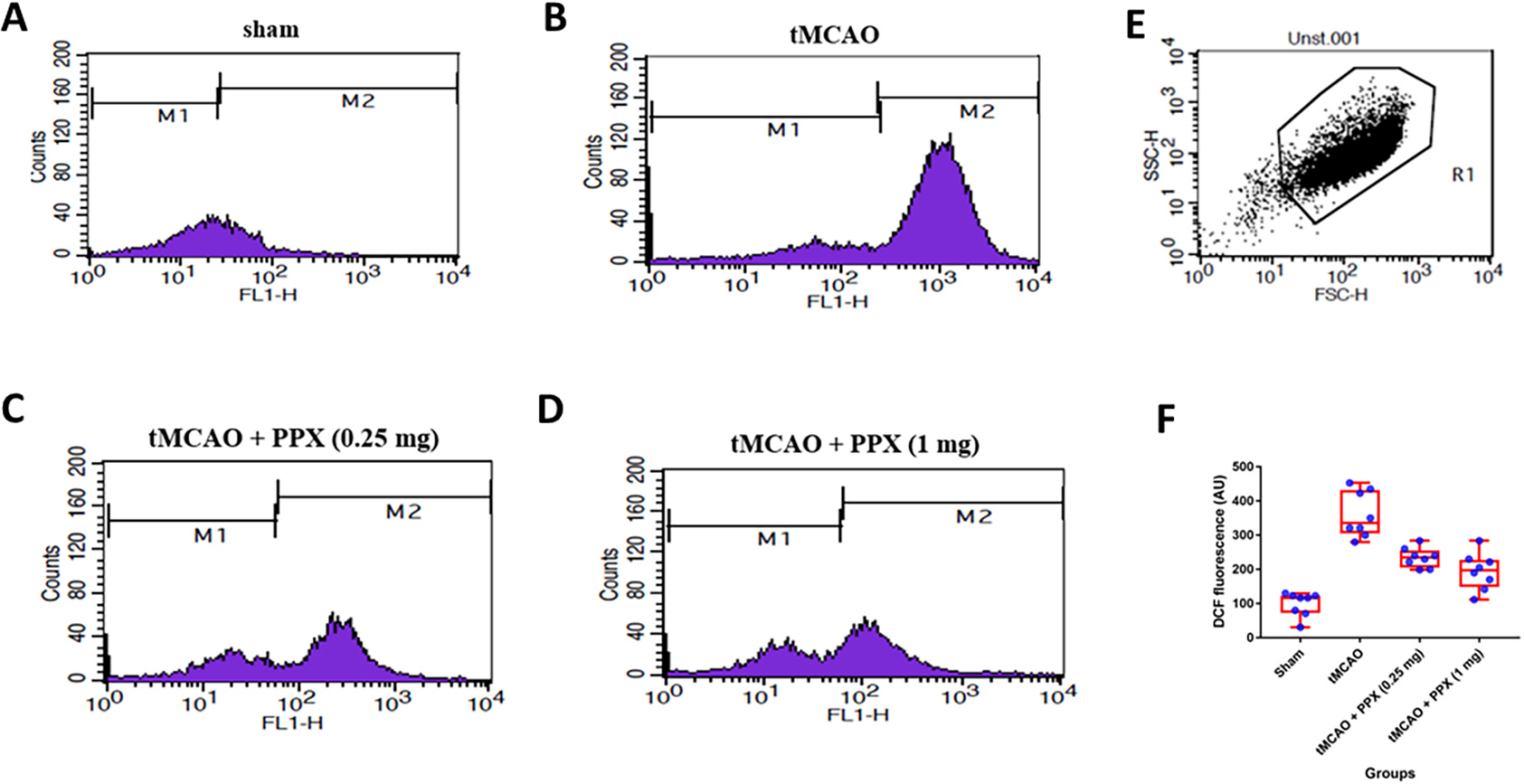
**PPX** **decreases levels of** **mitochondrial** **ROS.** (A-D) DCF fluorescence of mitochondria isolated from sham, tMCAO, tMCAO+PPX (0.25 mg/kg b.w.) and tMCAO+PPX (1 mg/kg b.w) rats. (E) In the FSC/SSC plot of the isolated mitochondria, 20,000 events were collected within gate R1. (F) Quantitative analysis of mitochondrial ROS. *P*<0.05 for tMCAO + PPX (0.25 mg/kg b.w.) versus tMCAO rats; *P*<0.01 for tMCAO + PPX (1 mg/kg b.w.) versus tMCAO rats.

The level of mitochondrial ROS was measured based on the intensity of dichlorofluorescein (DCF) fluorescence. Stroke induced a significant (*P*<0.001) increase in mitochondrial ROS as compared to sham animals. DCF fluorescence showed a significant difference (*F*_3,28_=39.80, *P*=0.0001) between groups. Post-stroke treatment with PPX significantly reduced the mitochondrial ROS as compared to stroke-only animals [0.25 mg/kg b.w. (*P*<0.05) and 1 mg/kg b.w. (*P*<0.01)] (Fig. 3A-F).

#### Mitochondrial calcium levels

**Fig. 4.**
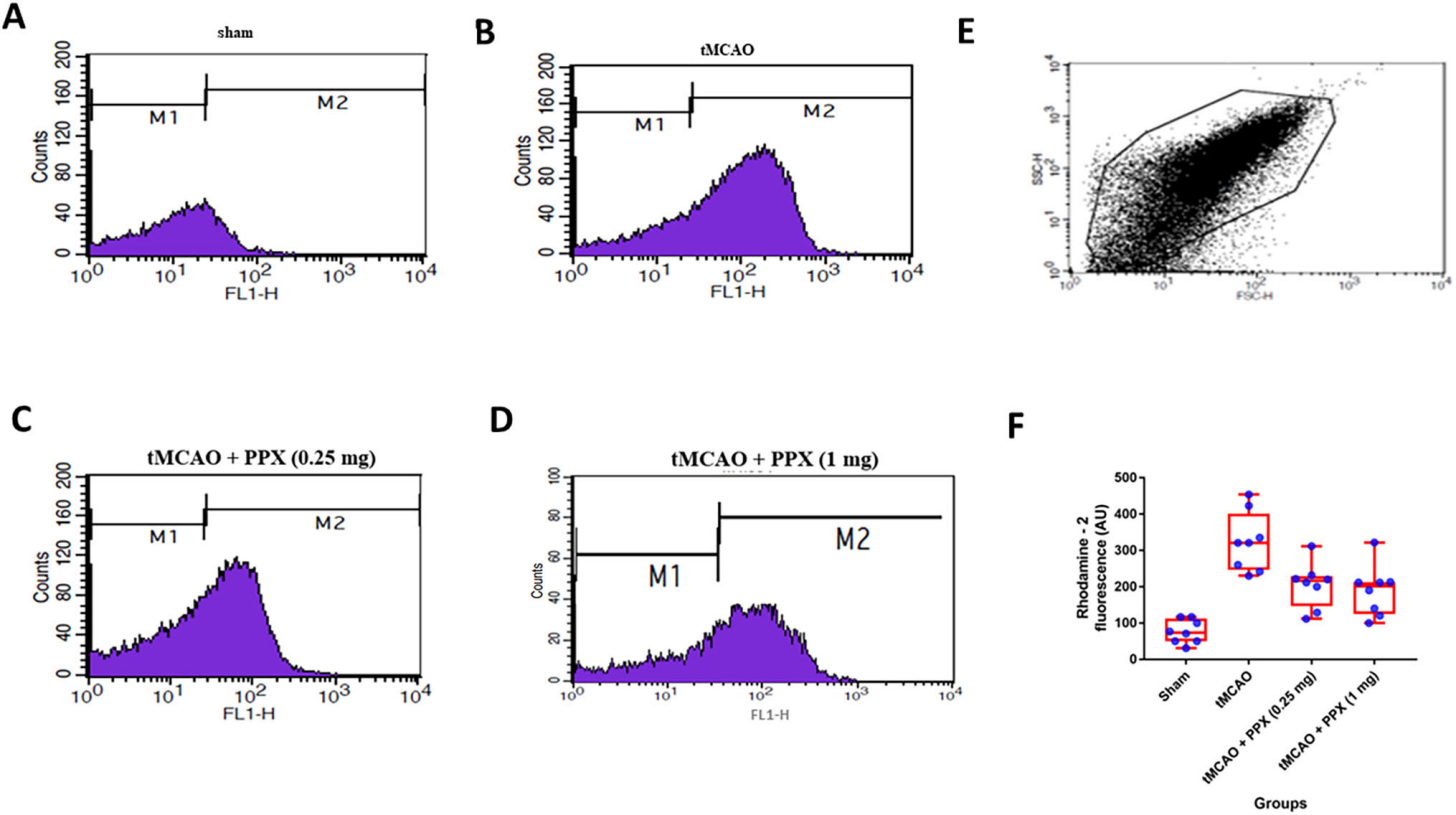
**PPX brings down mitochondrial Ca^2+^ levels.** (A-D) Rhodamine 2 AM fluorescence of mitochondria isolated from sham, tMCAO, tMCAO+PPX (0.25 mg/kg b.w.) and tMCAO+PPX (1 mg/kg b.w.) rats. (E) In the FSC/SSC plot of the isolated mitochondria, 20,000 events were collected within gate R1. (F) Quantitative analysis of mitochondrial Ca^2+^. *P*<0.05 for tMCAO + PPX (0.25 mg/kg b.w.) versus tMCAO rats; *P*<0.01 for tMCAO + PPX (1 mg/kg b.w.) versus tMCAO rats.

Stroke caused a rise in mitochondrial Ca^2+^ levels that was seen as a significant (*P*<0.001) increase in Rhodamine-2 fluorescence in tMCAO animals as compared to sham animals (Fig. 4A-F). There was a significant difference (*F*_3,28_=19.76, *P*=0.001) between groups (Fig. 4E). PPX treatment significantly reduced the mitochondrial Ca^2+^ levels as compared to tMCAO-only animals [0.25 mg/kg b.w. (*P*<0.05) and 1 mg/kg b.w. (*P*<0.01)] (Fig. 4A-F).

### Effect on mitochondrial membrane potential and swelling

**Fig. 5.**
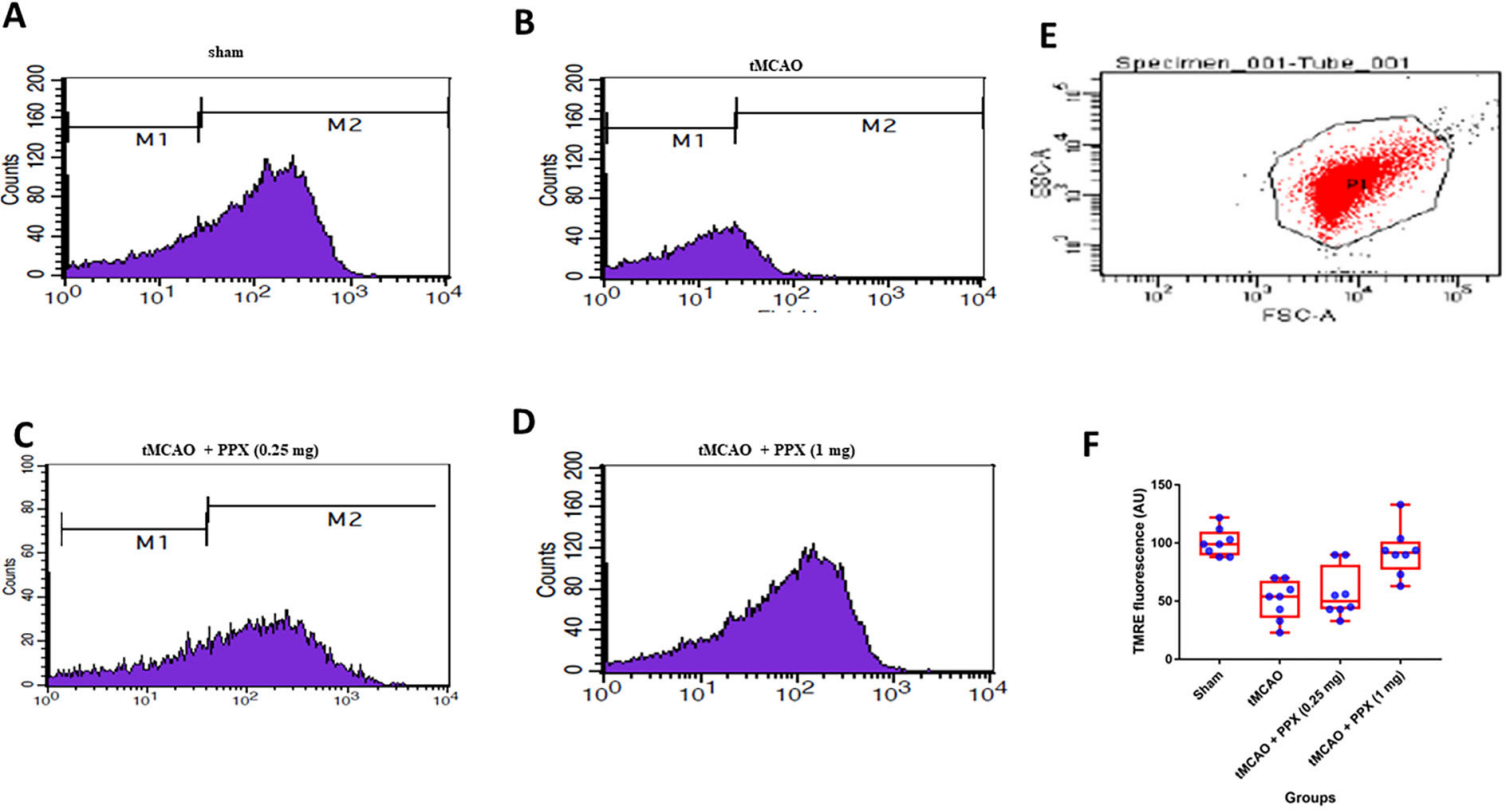
**PPX modulates** **the mitochondrial membrane potential.** (A-D) TMRE fluorescence of mitochondria isolated from sham, tMCAO, tMCAO+PPX (0.25 mg/kg b.w.) and tMCAO+PPX (1 mg/kg b.w.) rats. (E) In the FSC/SSC plot of the isolated mitochondria, 20,000 events were collected within gate R1. (F) Quantitative analysis of mitochondrial Ca^2+^. *P*<0.05 for tMCAO + PPX (1 mg/kg b.w.) versus tMCAO rats. No significant difference was found with lower dose of PPX.

**Fig. 6.**
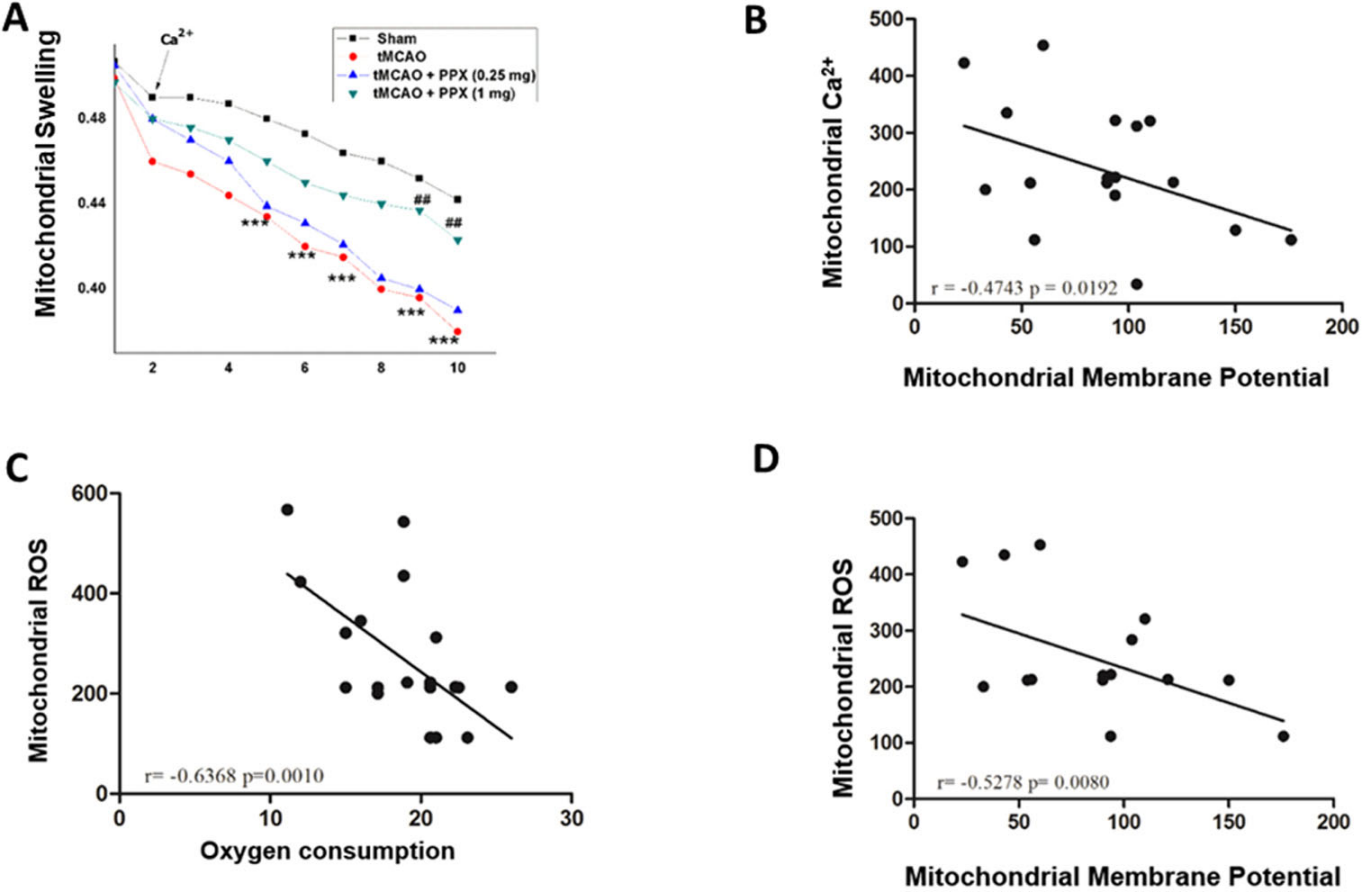
**PPX decreases mitochondrial swelling.** (A) Effect of PPX on mitochondrial swelling. In the tMCAO group, there was a significant (^##^*P*<0.001) decrease in the light absorbance as compared to sham rats. The PPX dose regimen of 1 mg/kg b.w. prevented the mitochondrial swelling significantly (***P<0.001). There was no effect of treatment with the lower dose of PPX (0.25 mg/kg b.w.). (B) Correlation between mitochondria Ca^2+^ and mitochondrial membrane potential. (C) Correlation between mitochondria ROS and oxygen consumption. (D) Correlation between mitochondria ROS and mitochondrial membrane potential.

The analysis of mitochondrial membrane potential was done by using a fluorescent probe of tetramethylrhodamine ethyl ester (TMRE). There was a significant difference (*F*_3,28_=15.05, *P*=0.001) between groups (Fig. 5E). PPX-treated rats showed a significant accumulation of TMRE inside mitochondria at the higher dose of 1 mg/kg b.w. (*P*<0.001), with no visible effect at the lower dose of 0.25 mg/kg b.w., as compared to tMCAO-only rats (Fig. 5A-F). The same result was observed in mitochondrial swelling experiments as there was less swelling in PPX-treated (1 mg/kg b.w.) rats as compared to tMCAO animals (*P*<0.001). There was no significant effect at a dose of 0.25 mg/kg b.w. (Fig. 6A).

### Effect of PPX on the mitochondrial oxygen consumption and respiratory control ratio

**Fig. 7.**
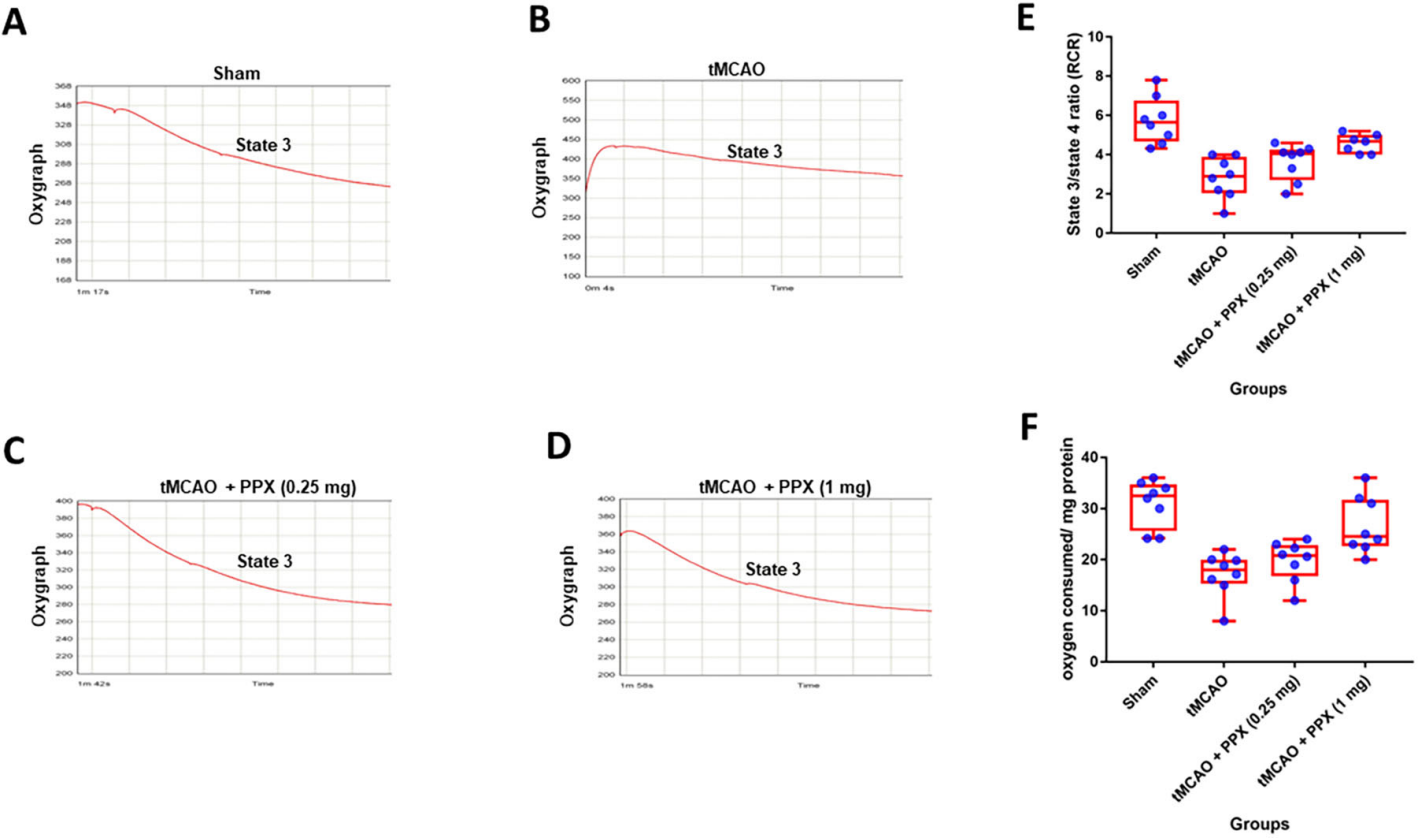
**PPX treatment modulates the mitochondrial bioenergetics.** (A-D) Oxygen consumption (ADP-induced state 3) of mitochondria isolated from sham, tMCAO- and PPX-treated animals. (E) There was a significant reduction in oxygen consumption (*P*<0.001) in tMCAO rats as compared to sham animals. PPX-treated animals showed a significant elevation in oxygen consumption (1 mg/kg b.w. *P*<0.001, 0.25 mg/kg b.w. *P*<0.05). (F) RCR (state 3/state 4) was also significantly (*P*<0.001) reduced in tMCAO rats as compared to sham group. PPX administration elevated the RCR significantly as compared to tMCAO rats (1 mg/kg b.w. *P*<0.001, 0.25 mg/kg b.w. *P*<0.05).

The respiratory control ratio (RCR) was affected in tMCAO animals (Fig. 7, Figs S1-S4). Oxygraph (oxygen electrode) showed a significant difference in both state 3 respiration (*F*_3,28_=14.86, *P*=0.001) and RCR (*F*_3,27_=13.72, *P*=0.001) between groups (Fig. 7E,F). PPX also elevated the oxygen consumption (*P*<0.001) in tMCAO-operated animals when compared to tMCAO-only rats (Fig. 7A-D). Both doses increased the oxygen consumption [0.25 mg/kg b.w. (*P*<0.05) and 1 mg/kg b.w. (*P*<0.01)] (Fig. 7E). Similarly, PPX treatment also increased the RCR in rats [0.25 mg/kg b.w. (*P*<0.01) and 1 mg/kg b.w. (*P*<0.001)] as compared to tMCAO-only rats (Fig. 7F).

### Effect on Bax:Bcl-2 ratio, Bax oligomerization and release of cytochrome *c*

**Fig. 8.**
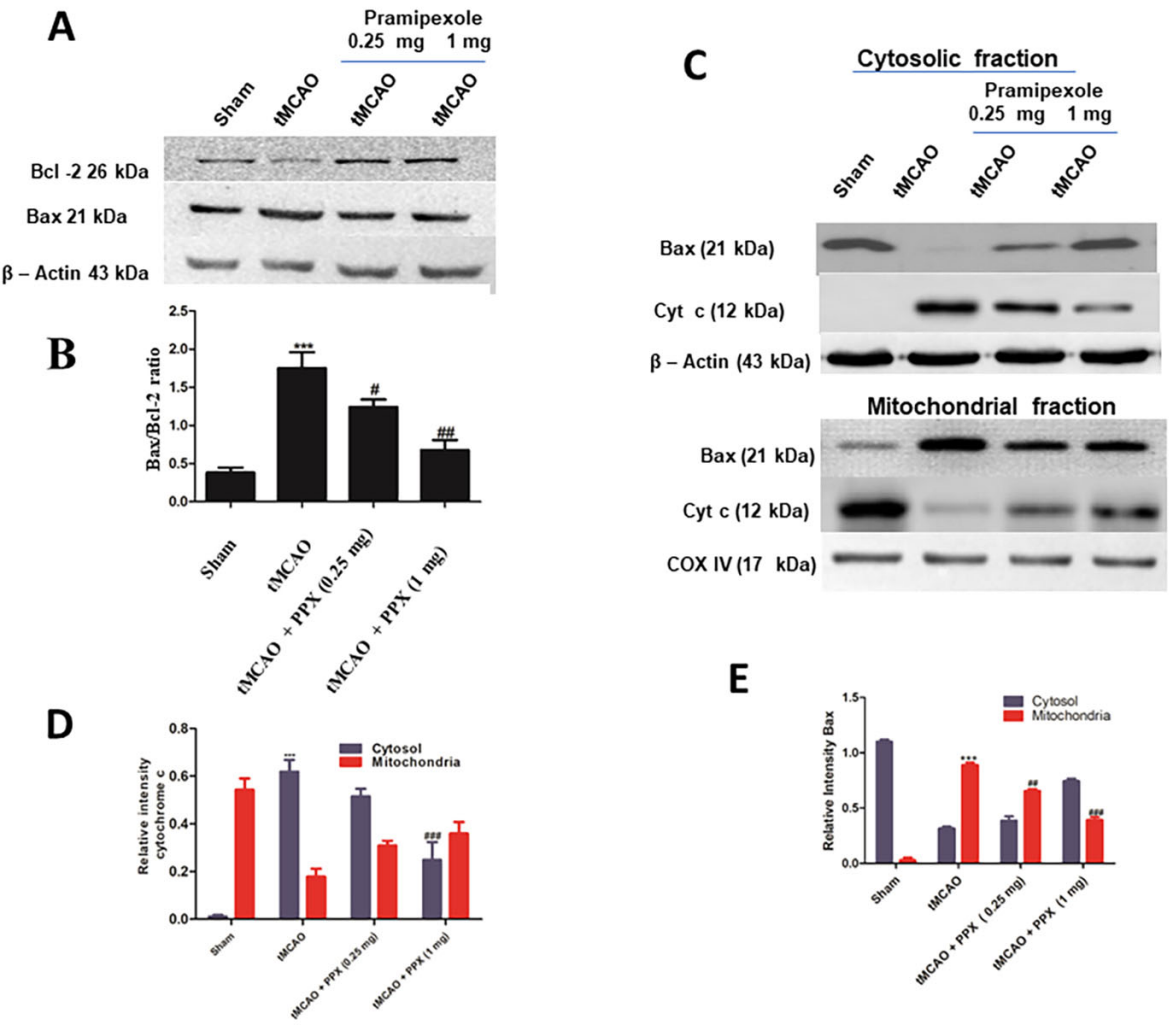
**PPX downregulates the Bax:Bcl-2 ratio** **and** **Bax oligomerization, and inhibits cytochrome** ***c*** **efflux to cytosol.** (A) Levels of Bcl-2 and Bax. (B) There was a significant (***P<0.001) rise in the ratio of Bax:Bcl-2 in tMCAO rats as compared to the sham group. PPX reduced the Bax:Bcl-2 ratio compared to tMCAO rats (1 mg/kg b.w. ^##^*P*<0.01, 0.25 mg/kg b.w. ^#^*P*<0.05). (C) Cytochrome *c* (cyt c) and Bax in mitochondria and cytosol. (D) There was a significant release of cyt c from mitochondria (***P<0.001) to cytosol in tMCAO rats as compared to the sham group. PPX inhibited the release of cyt c from mitochondrial to cytosol at a dose of 1 mg/kg b.w. (^###^*P*<0.001). There was no decrease in cyt c release from mitochondria to cytosol with 0.25 mg/kg b.w. of PPX. (E) There was significant oligomerization of Bax from cytosol to mitochondria (***P<0.001) in tMCAO rats as compared to the sham group. PPX inhibited decreases the Bax oligomerization from cytosol to mitochondria at a dose regimen of 1 mg/kg b.w. (^###^*P*<0.001) and 0.25 mg/kg b.w. (^#^^#^*P*<0.01).

The ratio of Bax:Bcl-2 can be used as an index of apoptosis. There was an increased expression of pro-apoptotic protein Bax in tMCAO-operated group as compared to sham animals (*P*<0.001) (Fig. 8A). PPX reduced the ratio of Bax:Bcl-2 with both doses [0.25 mg/kg b.w. (*P*<0.01) and 1 mg/kg b.w. (*P*<0.001)] as compared to tMCAO rats (Fig. 8A,B). Upon ischemia, Bax oligomerization takes place within the outer mitochondrial membrane (OMM), which increases the permeability of mitochondrial membrane. In tMCAO rats, Bax oligomerization increases, whereas PPX treatment decreased Bax oligomerization (Fig. 8C). In the tMCAO group, there was a significant increase (*P*<0.001) in the quantity of mitochondrial Bax as compared to the sham group (Fig. 8E). Post-stroke treatment with PPX also inhibited Bax oligomerization in mitochondria at both doses [1 mg/kg b.w. (*P*<0.001) and 0.25 mg/kg b.w. (*P*<0.01)] (Fig. 8E). Owing to the formation of mtPTP in tMCAO-operated rats, there was cytosolic release of cyt c from mitochondria to cytosol (Fig. 8C). In the tMCAO group, there was a significant increase (*P*<0.001) in the quantity of cytosolic cyt c as compared to the sham group (Fig. 8C). Post-stroke treatment with PPX also inhibited the release of cyt c from mitochondria at a dose regimen of 1 mg/kg b.w. (*P*<0.001). The dose of 0.25 mg/kg b.w. did not reduce the cyt c release from mitochondria as compared to tMCAO rats (Fig. 8D).

### Correlation analysis

A Pearson correlation analysis was used to evaluate the relationship between mitochondrial Ca^2+^ and mitochondrial membrane potential, between mitochondrial ROS and oxygen consumption, and between mitochondrial ROS and mitochondrial membrane potential (Fig. 6B-D). In addition, a separate Pearson correlation was used to analyze the relationship between the Bax:Bcl-2 ratio and cyt c release (Fig. 8E). A significant negative correlation was found between mitochondrial Ca^2+^ and mitochondrial membrane potential (*r*=−0.4743, *P*=0.0192) (Fig. 6B). We also found a significant negative correlation between mitochondrial ROS and mitochondrial membrane potential (*r*=−0.5278, *P*=0.0080) (Fig. 6D). Finally, there was a significant negative correlation between mitochondrial ROS and oxygen consumption (*r*=0.6368, *P*=0.6010) (Fig. 6C).

## DISCUSSION

Drug repurposing is a promising, fast and cost-effective strategy that can overcome traditional *de novo* drug discovery and developmental challenges of targeting neurological and other diseases. These drug repurposing tools and substantial experimental evidence can be applied to identify new pharmacological interventions for the existing drugs and can thus accelerate drug discovery (Strittmatter, 2014). In this context, dopamine agonists have shown great repurposing potential because of their immense neuroprotective properties (Takahashi-Yanaga, 2013; Winkelman et al., 2016). Previous studies have shown that PPX-promoted neuroprotection is associated with its anti-inflammatory, anti-apoptotic and anti-oxidative nature (Ferrari-Toninelli et al., 2010; Ma et al., 2016).

Mitochondria are the crucial regulators of cell survival in pathophysiological conditions such as ischemia/reperfusion (I/R) injury (Sims and Muyderman, 2010). The candidate drugs that can mediate their effect via mitochondria are gaining major attention and could be an effective tool for treating the disease (Choong and Mochizuki, 2017). Experimental evidence has shown that PPX induces the neuroprotective effect through the inhibition of mitochondria-mediated cell death (Rasheed et al., 2017). We have also previously demonstrated that PPX elicits neuroprotection by binding to the inner side of the mitochondria membrane that blocks the mtPTP by using patch-clamp recordings (Parvez et al., 2010; Sayeed et al., 2006). In the present study, we found that PPX improves neurological functions and reduces mitochondrial dysfunction when administrated 1 h post-occlusion in rats. The protective effects of the drug include improvement in the neurological functions, reduction in lesion volume and inhibition of mtPTP.

The neurological impairments were produced due to the critical damage of brain regions by I/R injury. Stroke-induced gait impairment is one of the clinically relevant indicators of motor dysfunction (Gama et al., 2017). In ischemic rats, severe neurological deficits were present. In addition, significant loss of movement was observed owing to damaged motor neurons in the frontal cortex region of the brain. Administration of PPX after ischemia inhibits the loss of neurons, reducing these neurological deficits. The loss of motor neurons, particularly in the primary cortex region, was observed to impair the motor coordination and grip strength post-stroke. Our data shows that PPX was able to improve the motor strength in tMCAO rats. In another panel of behavioral experiments, stroke-induced rats were slower to begin adhesive tape removal due to damage in the somatosensory cortex. PPX treatment might reduce the damage to the somatosensory region, resulting in the increased responsiveness of the rats in removing the adhesive tape from the affected limb. The staining of coronal sections with TTC showed a higher number of viable cells in the cortex of PPX-treated rats. Several reports suggest that activation of cell survival pathways and inactivation of apoptotic pathways might be a possible mechanism for inducing the neurological recovery (Nair and Olanow, 2008). Furthermore, administration of PPX reduced the gait impairment in the tMCAO rats. These findings are consistent with the previous studies showing that PPX reduces motor symptoms in rodent models of Parkinson's disease (Motyl et al., 2018). Brain infarctions as observed in the coronal sections were reduced after administration of PPX and, subsequently, decreased infarction was noted. This attenuation of infarction volume in the cortex regions of the brain might have improved the neurological recovery. It has been proposed that the reduction of motor symptoms by PPX is due to the stimulation of D2 receptor, but the actual method still remains elusive (Zhang et al., 2015). The anti-apoptotic mechanism of PPX could also be one of the explanations for inducing the functional recovery. Mitochondrial ROS production is critical for the progression of neuronal diseases, including stroke with severe cognitive impairment (Golpich et al., 2017). Pharmacological interventions aimed at mitochondria could be the key for the neuroprotection, based on significant prevention from stroke-induced functional deficits. Along with neuroprotection of the brain cortex, which can explain the improvement in motor and cognitive functions, there is also evidence that interference with mitochondrial pathways is effective for neurological recovery.

Recent attention has been drawn towards the mtPTP, a high-conductance channel responsible for permeabilization of the inner mitochondrial membrane, a process that leads to depolarization and Ca^2+^ release. Transient openings may be involved in physiological Ca^2+^ homeostasis, while long-lasting openings may trigger brain cell death after ischemia (Rizzuto et al., 2012). The influx of Ca^2+^ into the mitochondrial matrix was inhibited upon treatment with PPX. The potential inhibitory actions of PPX on the mitochondrial calcium uniporter could be a possible reason for this effect. Another mechanism behind the reduced influx of Ca^2+^ might be due to the antagonizing effect on the N-methyl-D-aspartate receptor (NMDAR) or the Ca^2+^ scavenging property of PPX, but the complete mechanism remains to be investigated thoroughly. Ischemia-promoted oxidative damage in mitochondria of brain cells leads to apoptotic and necrotic death of these cells. PPX decreased levels of mitochondrial ROS, thus providing protection from oxidative damage. These findings are consistent with previous studies that found that PPX induces neuroprotection via reduction of ROS (Ferrari-Toninelli et al., 2010). It is noteworthy that the triggering of a vicious cascade of mitochondrial damage could be due to the excessive accumulation of ROS and calcium in the mitochondria. This in turn may cause cyt c transport from mitochondria to cytosol, which could further promote mitochondrial-induced brain cell death (Akopova et al., 2012).

ROS increases intracellular calcium and enhances the formation of mtPTP, which leads to neuronal cell death in ischemic stroke. Induction of mtPTP swells the mitochondrial matrix and also dissipates the mitochondrial membrane potential (Wong et al., 2012). Previous experimental evidence has reported that, at physiological pH, PPX (being a doubly charged cation) can permeate through the plasma membrane of neuronal cells and bind the mtPTP at its negatively charged carboxylate groups (Luzardo-Alvarez et al., 2003). The lipophilic nature of PPX enhances its binding to both the cytosolic as well as the matrix side of the permeability pore (Sayeed et al., 2006). This could be one of the mechanisms of reversal of mitochondrial swelling and restoration of mitochondrial membrane potential. We found that inhibition of Ca^2+^ influx in the mitochondria matrix and excessive ROS production is also able to reverse the swelling and dissipations of membrane potential. There are strong negative correlations between the levels of mitochondrial Ca^2+^ and ROS with mitochondrial membrane potential, respectively.

The defective mitochondria persist in ischemic stroke, leading to enhanced oxidant production and oxidative injury as well as activation of oxidant signaling for cell death (Kalogeris et al., 2016). Our data showed a significant negative correlation between mitochondrial ROS and state 3 respirations, and vice versa. PPX induced the state 3 respirations and tightness of coupling between respiration and phosphorylation. Mechanistically, PPX promoted a reduction in mitochondrial ROS, which could be one of the possible explanations for these findings. Many studies support the anti-oxidative nature of PPX (Sayeed et al., 2006; Wang et al., 2018). Thus, it can be concluded that the anti-oxidative property of PPX could promote recovery in mitochondrial respiration after ischemia.

Bcl-2 family members regulate the release of proteins from the space between the mitochondrial inner and outer membrane to the cytosol. One Bcl-2 family pro-apoptotic protein, Bax, is usually localized in the cytosol or loosely attached to the mitochondria and undergoes conformational changes upon ischemia, leading to its translocation, oligomerization and insertion into the OMM. These Bcl-2-related proteins (Bcl-2, Bcl2l1, Bax and Bak) modulate the permeabilization of OMM in I/R-promoted neuronal death. We quantified the ratio of Bax:Bcl-2, which predicts the apoptotic nature of cells, by using western blotting. Although there was a decrease in Bcl-2 protein, elevation in the pro-apoptotic protein Bax was detected after ischemia. Administration of PPX significantly reduced the level of Bax protein in ischemic animals. These results are consistent with previous findings that demonstrated that PPX activates the PI3K/AKT/GSK3β pathway, which in turn inhibits Bax translocation to cytosol (Ma et al., 2016). Activation of AKT leads to the phosphorylation of downstream proteins, which are known to promote cell survival by inactivating pro-apoptotic proteins such as Bcl-2-associated death protein (BAD), Bax, caspase-9 and GSK-3β (Hermida et al., 2017). Our data shows that Bax oligomerization was inhibited by PPX treatment, suggesting an inhibitory role of PPX in Bax oligomerization. This could have decreased the permeabilization of the mitochondrial membrane. Here, we identified that PPX acts against Bax oligomerization in the OMM, which also inhibits apoptosis in brain cells. In previous studies, it has been demonstrated that a few small molecules can partially disrupt normal Bax and Bak dimerization at similar interfaces, thereby preventing dimers from forming higher-order oligomers, and thus established that proper Bax/Bak dimerization is necessary for OMM permeabilization (MOMP). Importantly, the group demonstrated that pharmacological inhibition of Bax and Bak with these small molecules allows cells to survive otherwise lethal stress and rescues neurons from prior excitotoxic damage (Niu et al., 2017). We used cytosolic cyt c as a marker of mtPTP activation or inhibition in our study. Mitochondrial events such as ROS production and Ca^2+^-induced swelling lead to cyt c release from mitochondria to cytosol, which in turn activates the apoptotic cascade, involving caspase-9. Our results showed a significant positive correlation between Bax:Bcl-2 ratio and cyt c release from mitochondria. Based on these findings, we can conclude that channel-forming proteins such as Bax affect the release of cyt c. PPX reduced the translocation of cyt c from mitochondria to cytosol. Previous studies have shown that PPX reduced cyt c translocation in various *in vivo* and *in vitro* models of PD (Shin et al., 2007). The mechanism behind the inhibition of cyt c could be explained in multiple ways. One of the mechanisms could be a reduction of mitochondrial ROS and Ca^2+^. Another mechanism might be the direct inhibition of mtPTP by the binding of positively charged PPX to negatively charged carboxylate groups of the mtPTP (Luzardo-Alvarez et al., 2003). Previously, it has been reported that, in rat liver mitoplasts, PPX directly inhibits the mtPTP in the inner mitochondrial membrane (Sayeed et al., 2006).

The clinical aspect of PPX to produce neurological recovery in stroke patients at this therapeutic dose is a critical issue. There are substantial arguments that PPX may be effective over a wide range of doses in PD patients. PPX has been found to be effective in reducing α-synuclein expression in PD patients at a dose of 0.25 mg (Luo et al., 2011).

Based on the current study findings, it can be concluded for the first time that PPX treatment produced neurological recovery through mitochondrial-mediated survival pathways in ischemic stroke. Therefore, PPX promotes mitochondrial recovery through the inhibition of mtPTP in an *in vivo* model of ischemic stroke of rat.

## MATERIALS AND METHODS

### Animals, surgical procedures, drug treatments and experimental design

#### Animals

All experiments and animals were approved by the Animal Ethics Committee, Jamia Hamdard, New Delhi, India. Male Wistar rats weighing 250-300 g (16-18 weeks old) were obtained from the Central Animal House Facility of Jamia Hamdard. Three to four animals were placed in the cage with a fixed light environment (12 h light/dark cycle), at 25°C and 40–60% humidity as well as free access to food and water.

#### Transient middle cerebral artery occlusion (tMCAO) model

We used transient cerebral ischemia induced by occlusion of the right middle cerebral artery (MCA) as previously described (Andrabi et al., 2017) with little modification. Prior to tMCAO surgery, animals were anesthetized with chloral hydrate (400 mg/kg b.w.). A midline incision was made on the ventral surface of the neck to expose the right common carotid artery. The external carotid artery (ECA) was ligated and internal carotid artery (ICA) was isolated near to bifurcation. An intraluminal monofilament of filament size 4.0, length 30 mm and diameter 0.19 mm, having a silicon rubber-coated tip, was introduced into the ECA and advanced through the ICA up to the origin of the MCA. The suture was withdrawn slowly after 2 h occlusion of the MCA, and rats were returned to their cages for the period of 22 h for reperfusion. In the sham group, the ECA was surgically prepared but the filament was not inserted.

#### Drug administration

The choice of dose and the route of drugs were made in agreement with the literature (Ma et al., 2016). PPX (A1237) at a dose of 0.25 and 1 mg/kg b.w. was dissolved in 0.9% ‘normal saline’, which was administrated by intraperitoneal (i.p.) injection 1 h post-occlusion and followed subcutaneously (s.c.) at 6, 12 and 18 h post-occlusion.

#### Experimental design

All the animals were trained for 6 consecutive days to get a baseline score for several neurobehavioral tests. At 24 h post-surgery, animals were tested for a number of behavioral tests then sacrificed for TTC staining.

The animals were divided in a randomized block design and the experimenter was blinded to the grouping of animals: (1) sham-operated group, (2) tMCAO group, (3) tMCAO+PPX (0.25 mg/kg b.w.), (4) tMCAO+PPX (1 mg/kg b.w.). All parameters were tested in the frontal cortex of the brain and *n*=6 were taken for each set of parameters in each group, respectively.

### Determination of neurological alterations

#### Assessment of neurological deficits

The neurological functions were recorded for 5 min in an animal cage based on a modified neurological severity scores (mNSS) scale (Zhang et al., 2016). Neurological deficits were assessed on a scale of 0-4 (0, no neurological deficit; 4, severe neurological deficit).

#### Motor impairment analysis

To assess the effect of PPX on motor impairment, the rats were evaluated in the rotarod task according to the previously described method (Andrabi et al., 2017). The score was presented as the mean of three trials for the duration (seconds) that the rat remained on the rotating rod.

#### Grip strength

To evaluate the forelimb grip strength, we used apparatus consisting of a string measuring about 50 cm in length, pulled tight between two vertical supports and elevated 40 cm from the flat surface. This method has been previously described (Tabassum et al., 2017).

#### Tape removal test

The tape removal test was used to assess the effect of PPX on sensory function according to the method described previously (Wali et al., 2014).

#### Gait pattern

Tests was done to find the gait-related anomalies at 24 h after tMCAO and were performed according to a previously described protocol (Trueman et al., 2017). SL and SW were taken as measures of gait parameters. SL is the length between fore paw and hind paw, whereas SW is the distance between the two forepaws.

### Assessment of infarction volume

At 24 h after surgery, the animals were sacrificed, and the brains were coronally sectioned into 1.5-mm-thick sections in a rat brain matrix and stained in 2% TTC solution before fixation in 10% formalin overnight. The infarction area was imaged with a scanner and analyzed using ImageJ (Wayne Rasband National Institute of Health, USA). Infarct size was calculated and expressed as a percentage by using the following formula to eliminate effects of edema as described previously (Zhang et al., 2016): [(contralateral volume)−(ipsilateral undamaged volume)]×100/(contralateral volume).

### Mitochondrial preparations

Differential centrifugation was used to isolate the mitochondria from frontal cortex of the brain according to the previously described method (Andrabi et al., 2017). Animals were decapitated and the frontal cortex was dissected and homogenized by using a mechanically driven Teflon-fitted Potter-Elvehjem-type homogenizer in ice-cold buffer A (see below). Mitochondria were isolated in three buffers: A, B and C. Buffer A contained 250 mM sucrose, 10 mM 4-(2-hydroxyethyl) piperazine-1-ethanesulfonic acid (HEPES), 1 mM EGTA,and 0.1% fat-free BSA adjusted by Tris to pH 7.4. Frontal cortex tissue was added to buffer A and centrifuged at 1000 ***g*** for 8 min at 4°C. The supernatant was collected and centrifuged at 10,000 ***g*** for 10 min at 4°C. Thereafter, the obtained pellet was resuspended and washed twice with washing medium (buffer B) containing 250 mM sucrose, 10 mM HEPES and 0.1 mM EGTA adjusted by Tris to pH 7.4, and centrifuged at 12,300 ***g*** for 10 min. Finally, the pellet was resuspended again in an isolation medium (buffer C) containing 250 mM sucrose, 10 mM HEPES and 0.1% fat-free BSA adjusted by Tris to pH 7.4, and centrifuged at 12,300 ***g*** for 10 min. The mitochondrial pellet was resuspended in buffer C, and the protein content was determined using the Bradford assay.

### Flow cytometric analysis of mitochondrial Ca^2+^, ROS and membrane potential

Flow cytometry analysis was performed using a FACSCalibur equipped with a 488 nm argon laser and a 635 nm red diode laser based on a prior methodology (Andrabi et al., 2017). The mitochondria were stained with tetramethylrhodamine, ethyl ester (TMRE; 100 nmol/l, excitation at 488 nm and emission at 590 nm), Rhodamine-2 (50 µm/l, excitation at 552 nm and emission at 581 nm) and 2′,7′-dichlorofluorescein diacetate (H2DCFDA; 10 mmol/l, excitation at 488 nm and emission at 525 nm), which were used to measure the changes of mitochondrial membrane potential, mitochondrial Ca^2+^ and the production of mitochondrial ROS, respectively. Mean fluorescent signal intensity was determined by flow cytometry to estimate mitochondrial ROS, Ca^2+^ and membrane potential.

### Ca^2+^-induced mitochondrial swelling

Mitochondrial permeability was assessed by Ca^2+^-induced mitochondrial swelling and was assayed spectrophotometrically as described previously (Andrabi et al., 2017). The aliquot of 100 µg of mitochondria was added to 1 ml of BSA-free and EDTA-free buffer, and 400 µm Ca^2+^ was added after 5 min and reading was taken for 5 min at 540 nm.

### Mitochondria respiration by oxygraph

Mitochondrial oxygen consumption was measured using a Clark-type oxygen electrode (Hansatech Instrument) based on a prior method (Waseem et al., 2016). The rate of mitochondrial oxygen consumption was measured as nanomoles of oxygen (O_2_)/min/mg of protein. The RCR was calculated as ratio state 3/state 4.

### Western blotting

The animals were decapitated and lysates were prepared according to the previously described method (Tabassum et al., 2017). The mitochondrial and cytosolic fractions were also prepared based on a previous method (Sanderson et al., 2013). The blotting was done on polyvinylidene fluoride membrane and the membranes were incubated with the primary antibodies (from Santa Cruz Biotechnology) anti-Bax (sc-493, rabbit polyclonal, 1:1000), anti-Bcl-2 (sc-7382, mouse monoclonal, 1:1000), anti-β-actin (sc-81178, mouse monoclonal, 1:1000), anti-cytochrome *c* (sc-13156, mouse monoclonal, 1:1000) and anti-COXIV (sc-292092, 1:1000) overnight at 4°C. The membranes were washed the following day with PBST and incubated with secondary antibody (HRP anti-rabbit IgG, 656120, goat anti-mouse IgG HRP, sc-2031, 1:10,000) for 2 h. After washing for 5-10 min with PBST, the detection of bound antibodies was visualized by chemiluminescence using the ECL-plus reagent. Anti-β-actin antibody was utilized to normalize protein loading and transfer. Densitometric analysis was performed by using ImageJ software (1.50 version, NIH, USA).

### Statistical analysis

All data were measured in terms of mean±s.e.m. All data were analyzed using GraphPad Prism 5 software (GraphPad Software Inc., San Diego, CA, USA). Behavioral data were analyzed using the one-way analysis of variance (ANOVA) followed by post hoc Tukey's test. The relative association between mitochondrial Ca^2+^ and mitochondrial ROS with mitochondrial membrane potential and oxygen consumption, respectively, were determined by Pearson correlation coefficient (*r*). Linear regression was determined to the strength of relationship among the parameters. Values of *P*<0.05 were considered significant.

## Supplementary Material

Supplementary information
