# Supplementary material for: Pramipexole prevents ischemic cell death via mitochondrial pathways in ischemic stroke
Source: Dis Model Mech. 2019 Aug 29;12(8):dmm033860. doi: 10.1242/dmm.033860 (PMC6737958; doi:10.1242/dmm.033860)
Supplement: Supplementary information [file dmm-12-033860-s1.pdf]

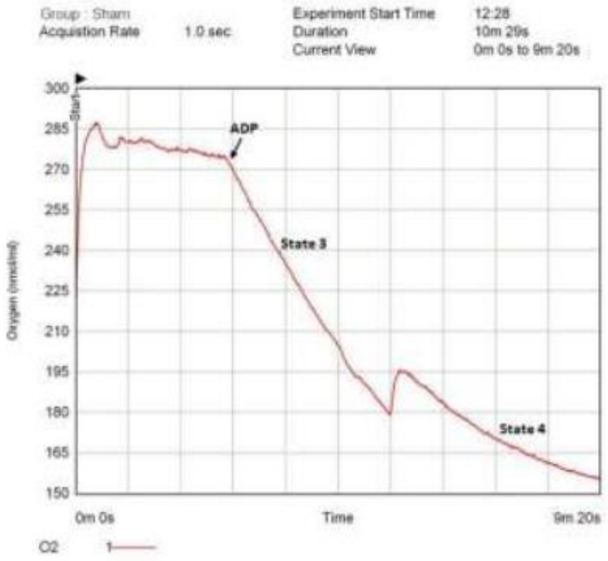

Fig.S1

Fig. S1. Represents the oxygraph images of sham groups with state 3 and state 4. Our data shows the significant reduction in oxygen consumption ($^{###}$P<0.001) in tMCAO rats as compared to sham animals.

Disease Models & Mechanisms • Supplementary information

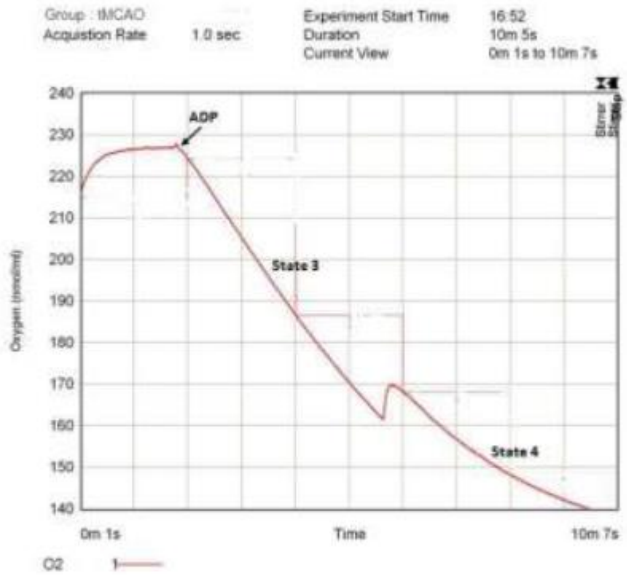

Fig.S2

Fig. S2. Represents the oxygraph images of tMCAO group with state 3 and state 4. Our data shows the significant reduction in oxygen consumption (###P<0.001) in tMCAO rats as compared to sham animals.

Disease Models & Mechanisms • Supplementary information

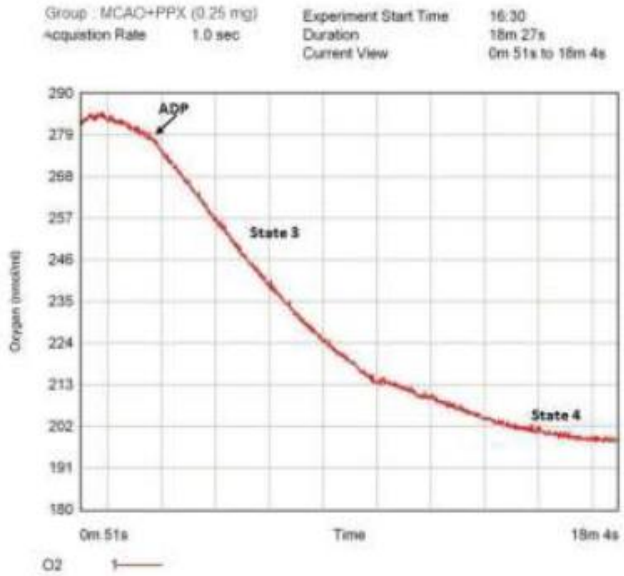

Fig.S3

Fig. S3. Represents the oxygraph images of tMCAO+PPX (0.25 mg/kg b.w.) group with state 3 and state 4. Our data shows the significant reduction in oxygen consumption (###P<0.001) in tMCAO rats as compared to sham animals. PPX treated animals showed significant elevation in oxygen consumption. (0.25 mg/kg b.w. #P<0.05). RCR (state 3/state 4) was also significantly (P<0.001) reduced in tMCAO rats as compared to sham group. PPX administration elevated the RCR significantly as compared to tMCAO rats. (0.25 mg/kg b.w. #P<0.05).

Disease Models & Mechanisms • Supplementary information

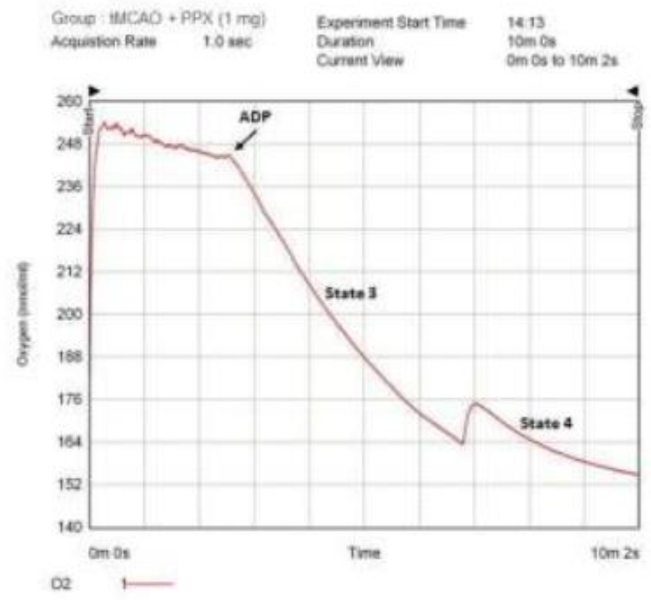

Fig.S4

Fig. S4. Represents the oxygraph images of tMCAO+ PPX (1 mg/kg b.w.) groups with state 3 and state 4. Our data shows the significant reduction in oxygen consumption (###P<0.001) in tMCAO rats as compared to sham animals. PPX treated animals showed significant elevation in oxygen consumption. (1 mg/kg b.w. ###P<0.001). RCR (state 3/state 4) was also significantly (P<0.001) reduced in tMCAO rats as compared to sham group. PPX administration elevated the RCR significantly as compared to tMCAO rats. (1 mg/kg b.w.###P<0.001).
